# Patient-Reported Outcome Measures in Pancreatic Cancer Receiving Radiotherapy

**DOI:** 10.3390/cancers12092487

**Published:** 2020-09-02

**Authors:** Ramez Kouzy, Joseph Abi Jaoude, Daniel Lin, Nicholas D. Nguyen, Molly B. El Alam, Ethan B. Ludmir, Cullen M. Taniguchi

**Affiliations:** 1 Department of Radiation Oncology, MD Anderson Cancer Center, Houston, TX 77030, USA; rkouzy@mdanderson.org (R.K.); Jbabi@mdanderson.org (J.A.J.); dlin4@mdanderson.org (D.L.); NDNguyen1@mdanderson.org (N.D.N.); mbel@mdanderson.org (M.B.E.A.); ebludmir@mdanderson.org (E.B.L.); 2 Department of Experimental Radiation Oncology, MD Anderson Cancer Center, Houston, TX 77030, USA

**Keywords:** patient reported outcomes, quality of life, pancreatic cancer, radiotherapy

## Abstract

**Simple Summary:**

Cancer therapies should improve patient survival or at least improve the quality of their life as they receive treatment for their disease. This is particularly important in pancreatic cancer, where current treatments often have to balance between limiting tumor growth and minimizing patient toxicity. There has been an increasing appreciation among physicians to capture the patient’s voice using tools called patient-reported outcome measures (PROM). In this article, we describe the available PROMs and their relative strengths and weaknesses to help oncologists make sense of this rapidly growing field. Finally, we present a decision-making tool that can help researchers and clinicians select the ideal PROM that fits their needs.

**Abstract:**

Pancreatic cancer and its treatment often dramatically impact patients’ quality of life (QoL). Given this, as well as increased focus on QoL measures in clinical oncology, there has been a rise in the number of instruments that measure patient-reported outcomes (PROs). In this review, we describe the landscape of different PRO instruments pertaining to pancreatic cancer, with specific emphasis on PRO findings related to pancreatic cancer patients receiving radiotherapy (RT). Twenty-five of the most commonly utilized PROs are compared in detail. Notably, most of the PRO tools discussed are not specific to pancreatic cancer but are generic and have been used in various malignancies. Published findings concerning PROs in pancreatic cancer involving RT are also extracted and summarized. Among the measures used, the European Organization for Research and Treatment Cancer QLQ-C30 was the most commonly utilized. We recommend a careful selection of PRO measures in clinical pancreatic cancer research and care and encourage the use of a combination of symptom-specific and global QoL tools to more fully capture patients’ perspectives.

## 1. Introduction

Patient-reported outcomes (PROs) are measures directly obtained from patients, including but not limited to emotional well-being, general quality of life (QoL), and physical and psychological symptoms. Since their introduction in the late 1960s, PROs have gained more attention over the 30 years that followed. The utility of PROs is now widely acknowledged both in clinical practice and research. This trend is particularly striking in clinical oncology, where there are widespread efforts dedicated to improving value-based quality of life for cancer patients. Professional organizations like the American Society of Clinical Oncology (ASCO) and the European Society of Medical Oncology (ESMO) now recommend the inclusion of PROs as central endpoints in modern clinical trials [1,2]. These guidelines are driven, in part, by the importance of QoL endpoints as real meaningful target goals and by studies having demonstrated better survival when PROs were collected, potentially due to earlier interventions preventing downstream consequences [3,4]. Furthermore, the Food and Drug Administration (FDA) has been increasingly shedding light on the value and use of PRO data in labeling and regulations. This enables quality-of-life metrics to be taken into consideration for drug approvals, in addition to quantity-of-life metrics such as overall survival (OS) or disease-control-related surrogates, most notably progression-free survival (PFS) [5,6,7].

In oncology, clinicians often assess toxicity and adverse events (AE) using standardized lexicons, such as the Common Terminology Criteria for Adverse Events (CTCAE). While historically adopted and extensively used, CTCAE toxicity measures rely primarily on physician or provider-based assessments of patients’ health and well-being. Such assessments are useful and often essential in order to properly assess and follow-up on patients. However, numerous studies show that CTCAE results and PRO results are not always congruent [8,9]. Contrary to the CTCAE, PRO data rely solely on the patient’s report. When studied further, data show that PROs capture the patient perspective in a complimentary manner to the clinician-based CTCAE report [8]. To illustrate this relationship, the National Cancer Institute (NCI) developed the patient-reported outcomes version of the CTCAE (PRO-CTCAE) to complement the clinician-reported CTCAE and aid in systematically capturing a wide spectrum of adverse events [10]. In light of proven differences between patient- and physician-based adverse events grading, experts call for the incorporation of both CTCAE and PRO in cancer clinical trials, in order to enhance the accuracy of adverse event reporting.

The standard methodology of acquiring patient-reported outcomes is through a questionnaire answered directly by the patient. The tools or instruments used to collect PROs are referred to as patient-reported outcome measures (PROMs). The precise methodology behind the development of PROMs varies. Generally, PROMs are designed based on adverse events that could be appropriate for patients to self-report. Those PROMs test specific domains and are typically developed with input from a variety of multidisciplinary stakeholders, including patients, clinicians, and psychometricians (for proper psychological measurements). In PROMs, a domain is made out of a number of measurable items that collectively describe a specific function or perception.

Each PROM must be validated in a specific patient population and should be capable of capturing the clinical differences effectively. These tools can either be general or disease-specific. Generic scales have the capability to measure a variety of different outcomes without venturing into a specific disease or outcome, like general physical function, pain, or social function. Disease-specific scales are usually tailored to a particular disease or set of diseases; they have the capability to shed focused attention on specific issues that are relevant to a particular disease process and/or its associated treatment. For instance, patients undergoing radiation therapy for pancreatic cancer may experience gastrointestinal toxicities secondary to radiation fields targeting the abdominal area, with a predominance of symptoms related to bowel dysfunction and toxicity. In such cases, PROMs measuring salivary gland function or respiratory symptoms may yield less useful information. Fortunately, many instruments are readily available now and can allow a researcher to easily capture the PROs for any specific disease site. The use of such PROMs has the potential to improve symptom reporting and management, as well as the establishment of an environment of understanding between patients and physicians. PROs offer the treating physician the opportunity to gain insight into the patient’s experiences while complementing the clinician’s assessment [11].

Understanding the patient experience is critical in the treatment of pancreatic cancer, since patients tend to experience substantial disease-related morbidity compounded by treatment-related toxicity from therapies such as RT; major surgeries (e.g., Whipple procedures); and potent multi-agent chemotherapies such as FOLFIRINOX (leucovorin, fluorouracil, irinotecan, and oxaliplatin) [12,13]. Despite the numerous modern clinical trials in pancreatic cancer, prognosis remains poor, with five-year survival rates of less than 10%. The poor clinical outcomes of patients with pancreatic cancer are not projected to improve in the near future, despite an ongoing rise in cancer incidences [14]. Therefore, it is crucial to design adequate quality-of-life tools in this population, since quantity of life remains unacceptably low [15]. Unfortunately, there is a substantial knowledge gap in PROs for the various available treatments of pancreatic cancer, particularly radiotherapy. This partly stems from a rapidly growing body of knowledge concerning the availability of PROMs and a lack of standardization across the field, as well as an increased emergence of novel RT technologies for the treatment of pancreatic cancer—in particular, stereotactic body RT (SBRT). In this review, we identify and summarize the different PRO instruments currently available for use in clinical and research settings and highlight PRO results from pivotal radiation studies in pancreatic cancer. We suggest potentially promising PRO metrics for clinical trials involving emerging therapies, such as SBRT.

## 2. Patient-Reported Outcome Measures in Pancreatic Cancer

Many PROMs have been studied in pancreatic cancer patients. Each tool was adopted for a specific trial or a subpopulation of patients with this disease. This has led to the creation of multiple excellent PROMs with their own unique scope, domain, and psychometric properties. However, this creates a problem for a trialist or researcher who wishes to implement a PRO metric into their own study—how do you select the right one?

The ideal PROM is brief but able to capture the symptomatology relevant to the disease of interest. Table 1 offers an overview of the available PROMs that have been utilized in pancreatic cancer. Generic PRO instruments cover a wide range of symptomatology without focusing on a particular domain or scope. Among the generic PROMs used in pancreatic cancer are the Short Form 36 (SF-36), the Brief Pain Inventory (BPI), and the Spitzer Quality of Life (QLI) tools. The SF-36 and its shorter version, the SF-12, remain widely used and are easy to both implement and interpret. Other PRO measures like the European Organization for Research and Treatment of Cancer Quality of Life Questionnaire Core 30 (EORTC-QLQ-C30), the Functional Assessment of Cancer Therapy-General (FACT-G), the MD Anderson Symptom Inventory (MDASI), and the EuroQoL 5Q cover broad areas like functional, physical, emotional, social, and cognitive domains. As such, these scales have been widely used, validated, and translated into various languages; however, they may not all be relevant across contexts and patient populations in oncology [16].

The use of cancer-specific measures like the EORTC or FACT-G, however, are thought to better capture the symptoms of oncological diseases and their treatments [17]. Both EORTC and FACT-G have great utility in cancer studies, since they can be broken into symptoms that focus on a particular cancer or group of malignancies, such as the EORTC-PAN26 and Pancreatic Cancer Disease Impact (PACADI) for pancreatic cancer. Of note, the EORTC-PAN26 has been found to be conceptually relevant to pancreatic cancer patients and tends to cover all concerns that the patients may experience, except neuropathic pain [18].

The importance of the patient perspective is growing in clinical trial design and endpoint selection. To that end, the FDA has established a list of core concepts regarding what a PRO measure(s) should assess [43], which includes symptoms related to disease and treatment, practical physical abilities, and standard adverse event reporting. PRO data generated from clinical trials help to define tolerability, safety, and the overall patient benefit of any intervention. Furthermore, the FDA now allows pharmaceutical companies to submit PRO data as a supplement for regulatory and labeling decisions, which can help approve novel medications that offer benefits in terms of quality of life and outcomes. As evidenced by Table 1, there exist a multitude of domains that cover cancer symptomatology but only a handful that are related to upper abdominal malignancies like pancreatic cancer. Identifying the PROM that captures the outcomes of interest is of paramount importance. Investigators and clinicians need to determine whether the measurement they are choosing adequately reflects their concept of interest.

**Figure 1:**
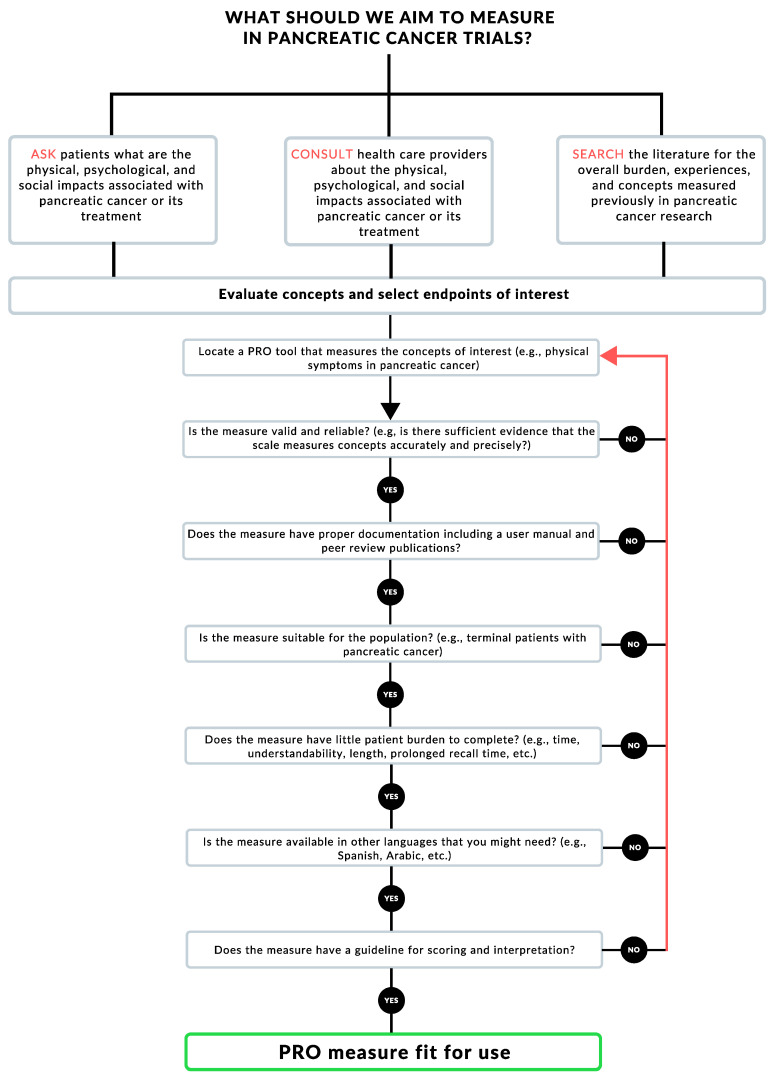
Patient-reported outcome (PRO) measure selection algorithm.

In Figure 1, we offer a simplified algorithm that can help pancreatic cancer researchers and trialists in selecting PROMs based on the research question they are interested in answering. After surveying and consulting with stakeholders on the endpoints of interest, researchers should locate and select a relevant PROM. For example, a researcher or clinician studying the side effects of a pancreatic cancer therapy might want to measure gastrointestinal symptoms, as well as general well-being. They would then locate a PROM that would measure both, such as the MDASI-GI. After locating a tool, the clinician/researcher would comb the literature to study if the PROM is valid and reliable in the population and setting of interest. The researcher would then go on to check each box in Figure 1; if the answer is satisfactory, they would move along the algorithm. If, on the other hand, the located PROM does not comply with any of the steps along the algorithm, the researcher/clinician would have to locate another tool and start the process again.

## 3. PRO Measures in Pancreatic Cancer Studies Using Radiotherapy

Surgery is the only known way to cure pancreatic cancer, and there have been several studies examining patient experiences with the Whipple procedure. Since surgery is not possible in most forms of pancreatic cancer (nearly 85% of patients, including patients with metastatic or locally advanced disease), RT is the only tested option to achieve local control. Definitive chemoradiation to pancreatic cancer is challenging because these tumors often grow into and around the nearby gastrointestinal tract, which cannot tolerate high doses of radiation. A highly conformal technique called stereotactic body radiation therapy (SBRT) delivers focused doses of radiation to the tumor, which results in better control with less injury to highly susceptible organs near the pancreas. Early SBRT studies for pancreatic cancer yielded unacceptable toxicity, but subsequent refinement of the technique has SBRT greatly improved and better local control and QoL [44,45,46,47,48] compared to conventional RT. Some studies suggest that this approach may synergize with immunotherapy or be dose-escalated with intensity-modulated radiation therapy (IMRT) or carbon ions.

The potential promise of radiation therapy in pancreatic cancer has been met with concerns over side effects and QoL, which has led investigators and clinicians to incorporate PRO measures in trials where radiation therapy was used. Table 2 highlights pancreatic cancer studies where QoL data were measured for radiation therapy and great heterogeneity of PRO data. In general, the use of RT pointed to a trend of better QoL without excessive toxicity, but an overarching conclusion cannot yet be reached, because PROs were not uniformly applied. The PROMs used in these pancreatic cancer studies with radiation varied widely from generic tools like the SF-36 to pancreatic cancer-specific tools such as the EORTC-QLQ-PAN26. In reporting the results of a phase 2 trial of SBRT for pancreatic cancer patients, Herman et al. utilized the EORTC-QLQ-C30/PAN26 to demonstrate that the addition of SBRT did not change patients’ global QoL (possibly a reflection of limited/minimal toxicities from SBRT) while improving patients’ pain (likely a reduction in tumor-related pain) [49]. This further highlights the importance of PRO measure selection in the study design and its ability to capture the intended changes in this specific population.

EORTC-QLQ-C30/PAN26 and FACT-Hep were the two most commonly used tools among pancreatic cancer trials [18] (Figure 1). These two PROMs are likely to be useful across a spectrum of pancreatic cancer trial designs, since these tools measure concepts of interests—in this case, cancer-specific symptomatology linked directly to pancreatic cancer. These tools have been validated and shown to be reliable, with a body of literature supporting their utilization. Additionally, both tools are accompanied by documents dictating the best practices, dealing with missing data, and analyses. The EORTC-QLQ-C30/PAN26 and FACT-Hep are validated and reliable in the pancreatic cancer population, with a similar time burden of 10–15 min (Table 1), which is reasonable. Additionally, both tools have a recall period of one week, which captures changes early enough while not burdening the patients with such a long recall period that they might cause undue difficulty recalling symptoms effectively. Moreover, both tools are available in multiple languages, which increases access to patients. Finally, these PROMs are supported by manuals for scoring and interpretation that can be found on their corresponding websites. In summary, these tools are tailored and fit for measuring PROs in pancreatic cancer trials and should be recommended for future utilization.

## 4. Conclusions

The importance of assessing the patient’s viewpoints through PROs is now recognized as critical data for patient care and trial design. Thus, increasing efforts are being dedicated toward incorporating PRO measures in clinical studies. The insights that these data provide can have profound effects on therapeutic decisions, since life expectancy with this disease remains dismal. Balancing QoL with therapeutic efficacy will likely have the most impact on patient satisfaction and clinical outcomes by personalizing care for each patient.

However, challenges remain in adopting the best practices into clinical trials, since there is no consensus with regards to the best PROM for a given study. Like any individual patient or treatment, the PROM must be tailored to data that would likely be relevant given the known morbidity of the cancer and its associated treatments. Among pancreatic cancer patients, data that can be generalized is of paramount importance, hence the underlying challenge of achieving a standard or benchmark that would allow clinicians and investigators to compare across studies and modalities. Such benchmarks might be the choice of measures, time points assessed, analyses, and interpretational techniques. In the absence of a unifying approach, the best practice for investigators and clinicians aiming to incorporate PRO data into their studies is to be aware of the characteristics of the PRO tools they are selecting and whether a combination might be necessary.

While this review focuses primarily on PROMs that can be used in patients with pancreatic cancer receiving radiotherapy, most of these measures can be used in other kinds of interventions in pancreatic cancer (e.g., chemotherapy or surgery) depending on the domains of interest. For trials involving radiotherapy in pancreatic cancer, the combination of a general QoL measure with a disease-specific PROM (e.g., EORTC-QLQ-PAN26) should be employed for a comprehensive understanding of the patient experience and symptomatology. The data generated offer insights into the patient’s perspective and can help physicians offer tailored therapies with the patient’s concerns in mind. More importantly, PROs might help bridge the relationship between the physician and patient, leading to better communication and insight, which, in turn, would lead to better outcomes and care satisfaction.

## Figures and Tables

**Table 1:** Patient-reported outcome (PRO) instruments available for use in pancreatic cancer.

Instrument	Items	Average Time Needed	Population for Intended Use	Scope	Recall Period
BFI [19]	4 items	5 min	Cancer patients regardless of treatment status	Impact and severity of cancer-related fatigue	24 h
BPI [20] *	32 items	10 min	Patients with chronic or acute pain	Severity and impact on daily functions of cancer-related pain	24 h/ 1 week
BPI short form	9 items	5 min			
CARES [21]	59 items	30 min	Cancer patients regardless of treatment status	Functional, physical, emotional, social, and cognitive	1 month
Distress Thermometer [22]	1 visual item	1 min	Cancer patients regardless of treatment status	Distress level	1 week
	35 item problem list	3 min		Psychosocial and physical needs	
Edmonton Symptom Assessment Scale [23] *	9 items	5 min	Cancer patients regardless of treatment status	Psychosocial and physical needs	Dependent on clinical setting
EORTC-QLQ-C30 [24]	30 items	10–15 min	Cancer patients regardless of treatment status	Functional, physical, emotional, social, and cognitive	1 week
EORTC-QLQ-C15-PAL [25]	15 items	5–10 min	Cancer patients in palliative care	Functional, physical, emotional, social, and cognitive	1 week
EORTC-QLQ-PAN26 [26] *	26 items	10–15 min	Pancreatic cancer patients	Functional, physical, emotional, social, and cognitive	1 week
EuroQoL (5Q-5D-5L) [27]	5 items 1 visual scale	5 min	Cancer patients regardless of treatment status	Mobility, basic self-care, daily activities, and pain, discomfort	1 week
FACIT-F [28]	13 items	5–10 min	Patients with chronic fatigue	Impact and severity of cancer-related fatigue	1 week
FACT-Hep [29] *	45 items	10–15 min	Pancreatic and hepatobiliary cancer patients	Functional, physical, emotional, social, and cognitive	1 week
FHSI-8 [30] *	8 items	5–10 min	Pancreatic and hepatobiliary cancer patients	Functional, physical, emotional, social, and cognitive	1 week
FHSI-18 [31] *	18 items	5–10 min	Pancreatic and hepatobiliary cancer patients	Functional, physical, emotional, social, and cognitive	1 week
GIQLI [32] *	36 items	10–15 min	Patients with gastrointestinal diseases	GI symptoms, functional, physical function, social, and emotional	2 weeks
MQOL [33] *	17 items	10–30 min	Cancer patients regardless of treatment status	Functional, physical, emotional, social, and cognitive	48 h
MDASI [34] *	13 items	6 min	Cancer patients regardless of treatment status	Psychological and physical symptoms	24 h
MDASI-GI [35] *	24 items	5 min	Cancer patients with symptoms caused by gastrointestinal cancer and its treatment	Functional, psychological, and gastrointestinal physical symptoms	24 h
MSAS [36] *	32 items	15 min	Cancer patients regardless of treatment status	Psychological and physical symptoms	1 week
PACADI [37] *	8 items	<5 min	Pancreatic cancer patients	Psychosocial and physical needs	1 week
PRO-CTCAE [38]	124 items	20 items~3.4 min	Cancer patients in clinical trials	78 symptoms from treatment toxicities that can be selected to build custom forms	1 week
PROMIS [39]	4–8 items per symptom	5 min	People with health conditions	Global health, distress, physical symptoms, cognitive function, etc.	1 week
SF-36 [40]	36 items	10–15 min	People with health conditions	Global health, distress, physical symptoms, cognitive function, etc.	1 month/1 week
SF-12 [41]	12 items	2–3 min			
Spitzer Quality of Life (QLI) [42]	5 items	1 min	Terminally ill patients	Daily activity, perceptions, behavior, and support	1 week

* = Validated in the pancreatic cancer population, BFI = Brief Fatigue Inventory, BPI = Brief Pain Inventory, CARES = Cancer Rehabilitation Evaluation System, EORTC-QLQC30 = European Organization for Research and Treatment of Cancer Quality of Life Questionnaire Core 30, EORTC-QLQ-C15-PAL = European Organization for Research and Treatment of Cancer Quality of Life Questionnaire for Palliative Care, EORTC-QLQ-PAN26 = European Organization for Research and Treatment of Cancer Quality of Life Questionnaire for Pancreatic Cancer, FACIT-F = Functional Assessment of Chronic Illness Therapy-Fatigue, FACT-Hep = Functional Assessment of Cancer Therapy—Hepatobiliary Cancer, FHSI = Functional Assessment of Cancer Therapy—Hepatobiliary Symptom Index, GIQLI = Gastrointestinal Quality of Life Index, MQOL = McGill Quality of Life, MDASI = MD Anderson Symptom Inventory, MDASI-GI = MD Anderson Symptom Inventory for gastrointestinal cancer, MSAS = Memorial System Assessment Scale, PACADI = Pancreatic Cancer Disease Impact, PRO-CTCAE = Patient-Reported Outcomes version of the Common Terminology Criteria for Adverse Events, PROMIS = Patient-Reported Outcomes Measurement Information System, and SF-36 = Short Form Health Survey.

**Table 2:** PRO in studies of pancreatic cancer involving radiation therapy.

Author	Study Aim	PRO Measure	Outcome
Polistina et al. [50]	Assessment of treatment response, local control, downstaging, pain, and QoL in patients with unresectable locally advanced PDAC undergoing SBRT.	SF-36	No QoL difference between pretreatment vs. 3- or 6-month control follow-ups.
Quan et al. [51]	Phase 2 clinical trial evaluating efficacy and safety of induction chemotherapy, followed by stereotactic ablative radiation therapy in borderline resectable and locally advanced PDAC.	FACT-G	No QoL difference between pretreatment and post chemotherapy, SABR, or surgery.
Krempien et al. [52]	Phase 2 clinical trial evaluating Cetuximab and chemoradiation (IMRT) in locally advanced PDAC.	EORTC QLQ-C30, EORTC QLQ-PAN26	Not available.
Morak et al. [53]	Comparison between QoL in patients who underwent adjuvant CRT compared to those who did not.	EORTC QLQ-C30	Better QoL in patients who underwent neoadjuvant CRT vs. observation only.
Knaebel et al. [54]	Comparison between OS between adjuvant 5-fluorouracil, cisplatin, interferon alpha, and radiation therapy vs. folinic acid and 5-fluorouracil.	EORTC QLQ-C30, EORTC QLQ-PAN26, CES-D	Not available.
Herman et al. [49]	Phase 2 clinical trial evaluating gemcitabine and SBRT in patients with locally advanced unresectable PDAC.	EORTC QLQ-C30, EORTC QLQ-PAN26	Global QoL scores remained stable from baseline to after SBRT. Pain scores improved 4 weeks after SBRT.
Serrano et al. [55]	To determine QoL during and after neoadjuvant CRT and surgery for patients with PDAC.	EORTC QLQ-C30, EORTC QLQ-PAN26, FACT-Hep	After neoadjuvant CRT, a transient increase in GI symptoms and a decrease in physical functioning were seen. After surgical resection, most QoL domains returned to baseline.
Short et al. [56]	To determine QoL as part of a phase 2 trial using the 3D conformal CRT sandwich technique in PDAC.	EORTC QLQ-C30, EORTC QLQ-PAN26	CRT improved local symptoms while not worsening global QoL.
Katz et al. [57]	To compare the efficacy of preoperative 5-FU vs. 5-FU plus hypofractionated SBRT/HIGRT in borderline resectable PDAC, primarily focused on evaluating and estimating the 18-month OS rate.	PRO-CTCAE	Not available.
Haddock et al. [58]	Phase 2 clinical trial to determine the efficacy, toxicity, and effects on QoL of radiotherapy with gemcitabine and cisplatin for patients with locally advanced PDAC.	SDS LASA	No significant overall QoL difference between baseline and the last measurement. However, overall SDS scores indicated improved QoL (specifically insomnia, frequency of pain, and outlook). LASA pain scores improved.
Heras et al. [59]	To analyze the effect of RT with 5-FU vs. RT with gemcitabine on QoL in patients with unresectable pancreatic cancer.	EORTC QLQ-C30	Overall QoL for both arms with RT significantly improved notably for cognitive function, decreased fatigue, and reduced appetite loss.
Hurt et al. [60]	To report QoL in patients with locally advanced pancreatic cancer during and after treatment with Cap- or Gem-CRT.	EORTC QLQ-C30, EORTC QLQ-PAN26	QoL improved at the induction of CRT, experienced significant decline during CRT, and recovered after the end of CRT in patients. Slight QoL differences favoring Cap-CRT.
Loehrer Sr et al. [61]	To evaluate Gem-CRT vs. Gem alone in patients with localized unresectable pancreatic cancer to determine if radiation improves survival or provides additional QoL.	FACT-Hep	No statistically significant QoL differences between Gem-CRT vs. Gem alone from the baseline comparison beyond week 6. However, there was a statistically significant decline in QoL among participants within each treatment arm from baseline to week 6.
Moore et al. [62]	Phase III trial to evaluate the effects of the addition of Erlotinib with Gemcitabine in patients with unresectable, locally advanced, or metastatic pancreatic cancer.	EORTC QLQ-C30	No significant difference in QoL between both treatment groups, with the exception of worsening diarrhea in the Erlotinib + Gemcitabine group.
Neoptolemos et al. [63]	Randomized control trial to evaluate the role of adjuvant chemoradiation and chemotherapy in patients with resectable pancreatic cancer.	ESPAC-1 QoL Form (based on EORTC QLQ-C30)	Overall, QoL increased for treatment groups (adjuvant CRT and chemotherapy vs. null) over 3 months from the baseline.
Neoptolemos et al. [64]	Phase 3 trial to evaluate the efficacy and safety of the combination of gemcitabine and capecitabine vs. gemcitabine alone in patients with resected pancreatic cancer.	EORTC QLQ-C30	No significant effect in the longitudinal estimate of QoL by treatment group.
Oettle et al. [65]	To determine whether adjuvant gemcitabine post-resection of pancreatic cancer improves disease-free survival by 6 months or more.	Spitzer QL-Index	QoL improved in both groups, with no significant differences between groups at any time point.
Conroy et al. [66]	Phase 2 and 3 trials to explore the differences between 5-FU and single-agent gemcitabine as a first-line treatment in patients with metastatic pancreatic cancer.	EORTC QLQ-C30	No overall differences in QoL between treatment groups, except 5-FU initially had higher scores for diarrhea, or of QoL in the 5-FU group as compared with the gemcitabine group.

PDAC = Pancreatic ductal adenocarcinoma, SBRT = Stereotactic body radiation therapy, SF-36 = Short Form Health Survey, QoL = Quality of life, FACT-G = Functional Assessment of Cancer Therapy—General, SABR = stereotactic ablative body radiotherapy, IMRT = Intensity modulated radiation therapy, EORTC-QLQC30 = European Organization for Research and Treatment of Cancer Quality of Life Questionnaire Core 30, EORTC-QLQ-PAN26 = European Organization for Research and Treatment of Cancer Quality of Life Questionnaire for Pancreatic Cancer, CRT = Chemoradiation, OS = Overall survival, CES-D = Center for Epidemiologic Studies Depression Scale, FACT-Hep = Functional Assessment of Cancer Therapy—Hepatobiliary Cancer, GI = Gastrointestinal, HIGRT = Hypofractionated image-guided radiation therapy, PRO-CTCAE = Patient-Reported Outcomes version of the Common Terminology Criteria for Adverse Events, SDS = Symptom Distress Scale, LASA = Linear Analog Self-Assessment, and RT = Radiation therapy.
